# Consumption of Sugar-Sweetened Beverages by Polish Young Adults: A Preliminary Study on the Importance of Sugar Tax Familiarity and Health Effects

**DOI:** 10.3390/foods13223616

**Published:** 2024-11-13

**Authors:** Marta Sajdakowska, Marzena Jeżewska-Zychowicz, Jerzy Gębski, Artur Kiełb

**Affiliations:** 1 Department of Food Market and Consumer Research, Institute of Human Nutrition Sciences, Warsaw University of Life Sciences (SGGW-WULS), 159C Nowoursynowska Street, 02-787 Warsaw, Poland; marzena_jezewska_zychowicz@sggw.edu.pl (M.J.-Z.); jerzy_gebski@sggw.edu.pl (J.G.); 2 The Social Sciences, University of Economics and Human Sciences in Warsaw, Okopowa 59 Street, 01-043 Warsaw, Poland; artur.kielb@wp.pl

**Keywords:** sugar-sweetened beverages, sugar tax, consumers, health, obesity

## Abstract

Consumption of sugar-sweetened beverages (SSBs) contributes to the risk of developing overweight and obesity in children and adults. Thus, reducing free sugar is a globally recognized public health priority. The implementation of a sugar tax is one way of achieving this goal. This study aimed to investigate the relationship between familiarity with the sugar tax and its importance in reducing obesity, the perceived health consequences of SSBs, situations and reasons for consumption, and the frequency of SSB consumption. A cross-sectional study was conducted in May 2022 among 216 Polish adults (age 18–45). Hierarchical cluster analysis was used to identify three homogeneous clusters with regard to the perceived health consequences of consuming SSBs. A logistic regression model was used to verify the relationship between the frequency of SSB consumption (dependent variable) and other variables. Respondents with higher familiarity with the sugar tax (OR: 0.594; 95% CI: 0.42–0.85) and those in the “Unaware of disease” (OR: 0.437; 95% CI: 0.18–0.94) and “Disease-conscious” (OR: 0.484; 95% CI: 0.24–0.97) clusters were less likely to consume SSBs at least once per week than those in the “Moderately disease-conscious” cluster. Both the place of SSB consumption (i.e., restaurant—OR: 1.847; 95% CI: 1.14–3.64, work or university—OR: 3.217; 95% CI: 1.51–6.88, family home—OR: 2.877; 95% CI: 1.50–5.53) as well as a preference for their taste (OR: 4.54; 95% CI: 1.66–12.45) increased the chance of consuming SSBs at least once a week. In conclusion, it is necessary to continue educating the public about the health consequences of SSB consumption. The use of legislative measures (i.e., sugar tax) also contributes to reducing SSB consumption and can thus counteract the increasing obesity rate.

## 1. Introduction

Sugar-sweetened beverages (SSBs) are a substantial source of added sugars worldwide [1]. Early introduction of SSBs to the diet, as early as in childhood, may condition individuals to have the tendency to consume sweet foods and beverages in the following years of life [2]. SSBs are not neutral to the body’s functioning. Consumption of such beverages contributes to a higher energy value of the diet, which determines an increased risk of developing overweight and obesity in children and adults [3,4,5,6], dental caries [7,8], and hypertension [2]. Moreover, added sugars in beverages increase blood glucose and insulin levels, which may contribute to an increased incidence of type II diabetes [9,10,11] and a higher risk of cardiovascular disease (CVD), mortality, and all-cause mortality [12]. SSBs also contribute to a reduced supply of calcium and other nutrients necessary for the proper functioning of the human body [5]. Reducing free sugar is a globally recognized public health priority in this situation. Several efforts are therefore being made to achieve this goal, including education [13], raising prices, and introducing taxes on SSBs [14]. The introduction of taxes on SSBs aims not only to reduce the consumption of sweetened beverages but also to encourage food producers to reduce the content of added sugars [15].

High consumption of sugar-sweetened beverages [16] and public health problems [17], together with adopted health strategies [18,19], led to the introduction of a so-called ‘sugar fee’ in Poland in 2021 [20]. This so-called ‘sugar fee’ has all the characteristics of a tax; i.e., it is non-refundable, compulsory, general, free of charge, and paid in the form of money [21]. A specific feature of the Polish sugar tax is that it applies to beverages with added sugar, sugar-free drinks (i.e., added sweeteners), and those with added taurine and/or caffeine [22]. The official rationale for introducing this tax was to counteract the increase in energy drink consumption among young people and to stop the trend of inadequate nutrition [15].

As a result of the implementation of the sugar tax, similar to in other countries [23,24], the prices of SSBs rose (by 32%) compared to the previous year [7]. A continuous decline in SSB consumption has been observed [7,25]. However, global consumption remains high. As a result, some SSBs have been replaced by beverages with lower sugar content or those that are artificially sweetened and in smaller portions [26,27]. Thus, manufacturers have tried to improve the nutritional value of beverages, which would not have happened to such an extent without the introduction of the tax [28]. Changes to the market offer of sweetened beverages are intended to help reduce the harmful health consequences of consuming large amounts of sugar, such as obesity and non-communicable diseases [29,30]. Nonetheless, changing consumer behavior is challenging due to its multifarious determinants [31,32]. Strategies for changing consumer behavior need to focus on food availability and affordability, the acceptability of healthier food options, and the consumer’s knowledge and awareness [33].

Low consumer knowledge of sugar sources encourages higher consumption of sugar-rich foods, making it difficult to estimate the sugar content of products and identify ingredients as sources of sugar [34,35]. Furthermore, consumers often infer the healthiness of foods based on other food characteristics; for example, fruit nectars and juices, compared to fizzy drinks, are usually perceived as healthier [36] and as containing less sugar [35]. However, awareness of the harm to health discourages consumers from buying sugary drinks, especially for children [37]. After the introduction of the taxes, reported changes in beverage purchasing varied across countries [38]. The observed lower consumption of sweetened beverages does not necessarily reflect tax pricing mechanisms. Still, a consumer shift away from sweetened beverages may be a positive outcome [38]. Moreover, most consumers are not replacing sweetened beverages with increased diet drinks, possibly due to concerns about sugar substitutes [39]. Previous studies have not explained the consumption of sweetened beverages and changes in consumption by considering consumers’ familiarity with the sugar tax and the perceived health consequences of consuming these beverages together.

Therefore, this study aimed to explore familiarity with the sugar tax along with its importance in reducing obesity and the perceived health consequences of sugar-sweetened beverage consumption. The relationships between these two factors, situations in which SSBs are consumed, reasons for consuming SSBs, and the frequency of consumption were then explored. This approach will identify the importance of familiarity with the sugar tax and consumer awareness of the health consequences of consuming sweetened beverages in changing the frequency of SSB consumption.

## 2. Materials and Methods

### 2.1. Data Collection Process

This cross-sectional study was performed using an online survey. An online questionnaire was designed using Google Forms. The main study was preceded by a pilot study among 20 respondents aged 18–40 who purchased food and declared their consumption of SSBs. This stage enabled us to verify the correctness and understanding of the questions. Prior to the survey, all of the respondents were told that the data and opinions gathered through the survey were confidential and would be used for research purposes only. For the main study, convenience sampling was used. The survey distribution was mainly performed by email invitation and social media in May 2022, only among those willing to participate in the study. For this purpose, appropriate information was provided to survey the participants, allowing them to decide on their participation in this research study. The inclusion criteria were age (18–45), declaring participation or co-participation in food shopping in the household, and drinking sweetened beverages (participants were included if they made the following responses to questions: To what extent do you decide on the food products purchased in your household?; I am responsible for all food purchases; I am jointly responsible for food purchases; I am not responsible for food purchases at all. Do you drink sweetened drinks?; yes/no). It was assumed that people over 45 are less active on the Internet, making recruiting them for the study difficult. In addition, the inclusion criteria were gender (female or male) and education—at least secondary school. The exclusion criteria were being younger than 18 or older than 45, education lower than secondary school, lack of participation or co-participation in food shopping in the household, and not drinking sweet beverages. In the end, 216 people were qualified for the study sample.

### 2.2. Description of Questionnaire

The questionnaire used in the study covered aspects such as (1) the impact of sweetened beverage consumption on the occurrence of diseases or health conditions; (2) the frequency of SSB consumption; (3) the importance of the sugar tax in dealing with obesity in Poland; (4) familiarity with the sugar tax; (5) situations in which SSBs are consumed; (6) reasons for consuming SSBs; (7) socio-demographic characteristics.

- The health effects of SSB consumption were assessed based on respondents’ opinions on the occurrence of diseases or health conditions as a consequence of drinking SSBs. Diseases or health conditions included tooth decay, overweight, obesity, type II diabetes, high blood cholesterol, and insulin resistance. All the above items were presented in a randomized order. The answers to the question “In your opinion, what is the significance of sweetened beverages on the development of the diseases or health conditions indicated above?” were marked on a 5-point scale, where 1—very small; 2—rather small; 3—neither small nor large; 4—rather large; 5—very large.
- The frequency of SSB consumption was defined at two levels, i.e., ‘less than once a week’ and ‘once a week or more often’. The frequency of consumption defined as ‘less than once a week’ was worded as follows: “I consume SSB 1–3 times a month”. The frequency of consumption formulated as ‘once a week or more often’ included the following statements: “I consume SSB once a week”, “I consume SSB several times a week”, “I consume SSB once a day”, “I consume SSB several times a day”.
- Regarding the importance of the sugar tax in dealing with obesity in Poland, respondents expressed their opinion on the statement “The sugar tax is a good solution in the fight against obesity in Poland” with the following answers: yes, no, no opinion.
- Familiarity with the sugar tax was assessed based on the respondents’ opinions on 3 pairs of statements: (1) the maximum fee is PLN 2.4/PLN 1.2 for 1 L of drink; (2) the fee for sugar content below or equal to 5 g in 100 mL of drink is PLN 0.50/PLN 1; (3) the fee for each gram of sugar above 5 g in 100 mL of the drink is PLN 0.10/PLN 0.05. For each pair of statements, one answer is true and the other is false. During the analysis, a true answer was given 1 point, while a false answer was given 0 points. Then, the points for each respondent were added up, and the obtained result ranged from 1 to 3 points. The higher the score, the more familiar the respondent was with the sugar tax.
- The situations in which the respondents consumed SSBs were assessed using the question, “In what situations do you consume sweetened beverages most often?” The respondent could choose more than one answer from the following: while in a restaurant, while at university/work, during family celebrations, while meeting friends, while at home, and during parties. As a reason for SSB consumption, the respondent could choose more than one answer from the following: it is how I satisfy my thirst, they give me more energy during the day, it is part of a typical meal for me, I like their taste, they are a reward for my successes, and they reduce my stress/tension.
- The questionnaire included questions on socio-demographic characteristics, including questions on gender, age, education, and respondents’ place of residence.

### 2.3. Statistical Analysis

Descriptive statistics were used to present the socio-demographic characteristics of the study sample. Data were presented as a sample percentage (%) for categorical data or mean and standard deviation (SD) for continuous data. The normality of the distribution of continuous variables was assessed using the normality Kolmogorov–Smirnov test. The Chi-square test of independence, the Mann–Whitney U test, or the Kruskal–Wallis test was used to determine if there were differences between subgroups. The level of statistical significance was set to *p* < 0.05.

The health consequences of consuming SSBs, i.e., tooth decay, overweight, obesity, type II diabetes, high blood cholesterol, and insulin resistance, were used to group respondents according to their awareness of the relationship between consumption of SSBs and the consequences mentioned above. Using a hierarchical cluster analysis method, three clusters were identified comprising respondents with similar views on the association between SSB consumption and the occurrence of diseases or health conditions (Table 1).

Based on the results obtained, the identified clusters were named as follows: “Unaware of disease” (N = 41), “Moderately disease-conscious” (N = 96), and “Disease-conscious” (N = 79) (Table 1). Then, the identified clusters were profiled according to the socio-demographic characteristics of the respondents, the frequency of SSB consumption, the situations in which these beverages were consumed, and the reasons for their consumption, as well as respondents’ familiarity with the sugar tax.

A logistic regression model was used to verify the association between the frequency of sugar-sweetened beverage consumption (the dependent variable) and the following exploratory variables: familiarity with the sugar tax, awareness of the relationship between the consumption of SSBs and the occurrence of a disease or health conditions (cluster membership), opinions on the importance of the sugar tax in determining the occurrence of obesity, and situations and reasons for consuming SSBs. A crude version of the model was developed as well as an adjusted version (considering variables such as gender, age, place of residence, and education as confounders). In the adjusted model, none of the adjusting variables (gender, age, place of residence, education) were statistically significant. Therefore, the results of the crude model were presented in this study. Odds ratios (ORs) represented the probability of adherence to the group characterized by drinking SSBs once a week or more often. The reference groups (OR = 1.00) drank SSBs less than once a week, were not familiar with the sugar tax (0 pts.), had an awareness of the relationship between the consumption of SSBs and the occurrence of a disease or health conditions (“Moderately disease-conscious” cluster), had opinions on the importance of the sugar tax in determining the occurrence of obesity (answer “no”), and declared no situations and reasons for consuming SSBs (answers “no”). Only statistically significant variables at the significance level of α = 0.05 were included in the model. Wald’s test was used to assess the significance of ORs. The Hosmer and Lemeshow goodness-of-fit test, the explained variation of survival (Nagelkerke R^2^), and the correctness of predictions were used to assess the quality of the resulting model.

The statistical analysis used the SAS 9.4 statistical package (SAS Institute, Cary, NC, USA).

## 3. Results

### 3.1. Description of the Sample

The study participants were primarily women and people under the age of 25. No significant differences were found in the socio-demographic characteristics of the identified clusters (Table 2).

### 3.2. Characteristics of Identified Clusters

More than half of the respondents (56.9%) declared that a sugar tax is irrelevant in the fight against obesity in Poland, while about one-fifth agreed with this statement (21.3%). Opinions on the sugar tax as a good solution in the fight against obesity in Poland did not differ between clusters. There were also no differences in familiarity with the sugar tax between groups of respondents with similar views on the relationship between the consumption of SBBs and the occurrence of diseases or health conditions (clusters) (Table 3).

The frequency of SSB consumption differed between clusters (Table 3). The “Moderately disease-conscious” cluster presented the highest rate of participants who consumed SSBs at least once a week (59.4%), while the “Unaware of disease” cluster had the lowest rate (39.0%). SSBs were consumed by more respondents during meetings with friends (63.4%) and parties (61.6%), followed by during family celebrations (50.9%). About one-fourth of respondents consumed such beverages at home, and the fewest percentage of respondents consumed them at university/work (25.9%). More than four-fifths of the respondents (81.5%) consumed SBBs because of their taste, and for 19.5% of people, drinking such beverages was a way to quench their thirst (Table 3). There were no differences between clusters in terms of situations in which SSBs are consumed. However, most respondents from the “Moderately disease-conscious” cluster satisfied their thirst with SSBs, while in the “Disease-conscious” cluster, only 7.6% provided such an answer (*p* < 0.001).

### 3.3. Prediction of Frequent Consumption of Sugar-Sweetened Beverages

Consuming SSBs at least once a week was less likely in the “Unaware of disease” cluster (by 56.3%) than in the “Moderately disease-conscious” cluster (OR: 0.437; 95% CI: 0.18–0.94). Similarly, consuming SSBs at least once a week was less likely in the “Disease-conscious” cluster (by 51.6%) than in the “Moderately disease-conscious” cluster (OR: 0.484; 95% CI: 0.24–0.97).

Those who reported drinking SSBs while in a restaurant were 84.7% more likely to consume them at least once a week than those who did not drink them in these circumstances (OR: 1.847; 95% CI: 1.14–3.64). Among those drinking SSBs at work or university and at home, there was a three times higher likelihood of consuming them at least once a week compared to non-drinkers (OR: 3.217; 95% CI: 1.51–6.88; OR: 2.877; 95% CI: 1.50–5.53, respectively).

Respondents who declared that they drink SSBs because of their taste (“I like their taste”) were more than 4.5 times more likely to consume them at least once a week than those who did not declare such taste preferences (OR: 4.54; 95% CI: 1.66–12.45).

A higher score in familiarity with the sugar tax decreased the chances of consuming SSBs at least once a week. Each additional score point reduced these chances by 40% (OR: 0.594; 95% CI: 0.42–0.85) (Table 4).

## 4. Discussion

Based on the perceived health consequences of sugar-sweetened beverage consumption, we considered three clusters (“Unaware of disease”, “Moderately disease-conscious”, and “Disease-conscious”) to explore familiarity with the sugar tax along with its importance in reducing obesity and the frequency of sugar-sweetened beverage (SSB) consumption. The findings of previous studies on the relationship between health-related knowledge and SSB consumption are mixed [34,40,41,42]. The lack of such a relationship in some studies suggests that knowledge of SSB-related health conditions may not be sufficient for adult behavior change or that varying levels of knowledge have different impacts on SSB intake in diverse populations [42,43,44]. In our study, the frequency of SSB consumption differed between the identified clusters. Respondents who consumed SSBs at least once a week were mostly in the “Moderately disease-conscious” cluster. In the other clusters, i.e., the “Unaware of disease” and the “Disease-conscious” clusters, the proportion of people consuming SSBs with this frequency was lower. On the one hand, the previously demonstrated lack of association between knowledge of the consequences of consuming SSBs and frequency of consumption [43] could explain the lack of differences between those aware and unaware of the health consequences of consuming SSBs. Moreover, the frequency of consumption of SSBs may depend on many other factors, with awareness of the consequences of their consumption being only one [41]. Thus, among the “Moderately disease-conscious”, the preference for these products due to their taste may cause more frequent consumption [45,46]. The results of previous studies underline that taste-oriented motivations still predominate compared to health motivations when making food choices [47,48,49]. Moreover, paying little attention to or even ignoring health as a value [50] despite having knowledge may also influence behavior, which could explain the frequent consumption of SSBs in the “Moderately disease-conscious” cluster.

In the study group, the tax was not perceived as a good solution in the fight against obesity in Poland. However, taxation strategies are effective, as evidenced by the decreases in SSB sales in many countries [26,51,52,53,54], and they are also effective regarding dietary intake [55]. Moreover, SSB taxes increase the probability of choosing healthier beverages [3,53]. Generally, such taxes on sugar-sweetened beverages can increase welfare throughout society by encouraging consumers to limit unhealthy consumption [56]. One of the effects of the SSB tax can be the transmission of information about the health risks associated with SSBs. This signaling effect of the SSB tax should increase consumer health awareness, including obesity awareness [57]. Since no differences in familiarity with the sugar tax were found between the identified clusters, it can be assumed that this study’s results did not confirm this effect. However, opinions before and after the introduction of the tax were not compared. It can be assumed that the increase in the price of SSBs as a result of the sugar tax influences views on its ineffectiveness in reducing obesity, because price is a very important factor in their purchase [58,59,60].

SSBs were mainly consumed in social situations, i.e., when spending time at a restaurant, having family celebrations, meeting with friends, having parties, as well as when drinking beverages while staying home. Other studies confirm that drinking SSBs is very common while eating, especially during social events [61,62]. Moreover, consumers tend not to sufficiently reduce their intake of other foods to compensate for the extra calories SSBs provide [63]. The consumption of various meals accompanied by drinking SSBs during social events further increases the risk of weight gain [4]. A link has been demonstrated between the consumption of free sugars, particularly in the form of SSBs, and weight gain in both children and adults [6], while reducing the intake of SSBs has been shown to reduce weight gain in children, particularly in those who are already overweight [4]. Our study found that consuming SSBs only in places such as restaurants, homes, and workplaces increased the chance of consuming SSBs at least once a week.

Among respondents who declared that they drink sweetened drinks because of their taste, the likelihood of drinking them at least once a week was 4.5 times higher than for those who did not declare such taste preferences. Taste is the main reason for consuming sweetened beverages, which is unsurprising since humans prefer sweet tastes over other tastes. Highly palatable foods like SSBs might trigger regulating systems, leading to overconsumption [64]. Moreover, palatable foods (usually high in sugar, fat, and salt) taste more intense, and this may result in a stronger effect on rewarding mechanisms, leading to the release and/or up-regulation of reward mediators such as serotonin-, dopamine- and/or opiate-related pathways [65]. The global food supply has become sweeter in the past decades due to an increase in food products with added sugars or non-nutritive sweeteners [66]. The availability of such products encourages their consumption and thus the formation of preferences. In addition, childhood exposure to SSBs and sweet foods favors their consumption but can also contribute to sweet food preferences in later life [67]. To slow down these changes in preferences, producers should be encouraged to reformulate and produce low and no-calorie sweetener products, enabling consumers to adapt to the less sweet taste of beverages [68]. Moreover, reformulation can cause a decrease in beverage purchases [69], which can result in a lower level of consumption [24,55,70].

It should be emphasized that, apart from legislative actions, it is also important to educate society about the health effects of excessive sugar consumption, because a reduction in sugar intake is advised around the world as part of healthier dietary patterns to help reduce energy intake, obesity risk, and obesity-related disorders [71]. Other studies in the literature also indicate that for some food groups, including sugar-sweetened beverages, sugar content reduction could have the most significant impact on public health [72]. At the same time, it should be emphasized that the sugar tax can have a supportive effect on the education of society. This is all the more important, as our research found that the higher the familiarity with the sugar tax, the lower the chance of drinking sugar-sweetened drinks at least once a week. Other studies indicate that with increased knowledge about nutrition recommendations, consumers made beneficial nutritional decisions [73,74]. Moreover, education can increase nutritional knowledge and strengthen health motivation [75].

According to Koczkodaj and Michałek (2024), to fully harness the potential of this fiscal solution, consideration should be given to tightening regulations, especially regarding the value of fees, the categories of products they apply to, and the allocation of funds obtained through the sugar tax, which should predominantly be directed towards obesity primary prevention activities. Furthermore, the lack of detailed and publicly accessible reports on the allocation of funds from the sugar tax intensifies public discussion regarding the use of sugar tax revenues in Poland and the actual aim of its introduction. Due to this fact and the short duration of the sugar tax implementation in Poland, it is challenging, if not impossible, to estimate its current impact on the health of the Polish population. Nevertheless, the potential positive effects of the discussed tax on the public health sector are invaluable [76].

## 5. Strengths and Limitations

In previous studies on the relationship between the perception of the health consequences of sugar-sweetened beverage consumption and behaviors, researchers included variables describing single consequences of the consumption of SSBs in the analyses. The cumulative perception of the consequences of SSB consumption and its relationship to behavior can be more relevant in explaining SSB consumption. That is why we used cluster analysis to identify homogenous groups according to the perceived health consequences, which let us assess the cumulative perception of several health consequences of SSB consumption. This methodological approach provides more opportunities to use the results in developing educational and intervention programs. Besides this strength mentioned above, this study has some limitations. One of them is that the study relied on self-reported information. Consuming SSBs may be perceived as acceptable or unacceptable depending on social group, so responses regarding the frequency of consumption may reflect social expectations. This study was conducted using a convenience sample, which does not allow for generalizations regarding society, even regarding the population of young Poles. The sample size (N = 216) and the predominance of females and participants under 25 years of age should be treated as limitations of this study. This study focused on selected motives for consuming SSBs, i.e., familiarity with the sugar tax and its importance in reducing obesity, perceived health consequences of SSBs, and situations and reasons for consumption. Likewise, other motives should be considered in further studies, such as taste, price, etc.

## 6. Conclusions

A higher familiarity with the sugar tax and being unaware or aware of disease as a consequence of consuming SSBs were predictors of consuming SSBs less frequently. Drinking SSBs when in a restaurant, at work/university, at family celebrations, or at home increased the chance of consuming SSBs at least once a week. Moreover, a preference for their taste favored their consumption at this frequency.

This study’s findings indicate that it is necessary to undertake new programs and continue existing initiatives to educate the public, especially those unaware of the health consequences of sugar-sweetened beverage consumption. At the same time, applying legislative measures such as a sugar tax can contribute to limiting the consumption of these products. Still, action is also needed to increase consumer awareness of these legislative measures. However, further research is needed, especially in representative groups and taking into account other factors influencing the consumption of SSBs, which help create a better understanding of consumer behaviors, especially of those who are moderately disease-conscious.

## Figures and Tables

**Table 1:** Clusters identified based on the perceived importance of SSB consumption in causing diseases or health conditions.

Diseases and Health Issues	Cluster 1 “Unaware of Disease” (N = 41)	Cluster 2 “Moderately Disease-Conscious” (N = 96)	Cluster 3 “Disease-Conscious” (N = 79)	*p*-Value *
Tooth decay	33.3 c **	104.3 b	152.7 a	<0.001
Overweight	31.3 c	100.5 b	158.3 a	<0.001
Obesity	32.2 c	99.9 b	158.5 a	<0.001
Type II diabetes	32.2 c	97.0 b	162.0 a	<0.001
High blood cholesterol	52.0 c	88.7 b	161.9 a	<0.001
Insulin resistance	39.7 c	88.9 b	168.0 a	<0.001

* Kruskal–Wallis test. ** Mean rank, a, b, c—different letters indicate significant differences between the values in the row.

**Table 2:** Socio-demographic characteristics of consumers (%).

Items		Total Sample	Cluster 1 “Unaware of Disease” (N = 41)	Cluster 2 “Moderately Disease-Conscious” (N = 96)	Cluster 3 “Disease-Conscious” (N = 79)	*p*-Value *
Gender	male	26.4	39.0	27.1	19.0	0.060
	female	73.6	61.0	72.9	81.0	
Age (in years)	up to 25	75.1	78.0	81.1	66.2	0.073
	25–45	24.9	22.0	18.9	33.8	
Education	secondary	56.0	58.5	61.5	48.1	0.195
	higher	44.0	41.5	38.5	51.9	
Place of residence	rural area	21.8	19.5	26.0	17.7	0.170
	city of under 20,000	7.4	7.3	2.1	13.9	
	city of 20–100,000	13.9	17.1	14.6	11.4	
	city of 101–500,000	16.2	17.1	13.5	19.0	
	city of over 500,000	40.7	39.0	43.8	38.0	

* Chi^2^ independence test.

**Table 3:** Selected opinions on the sugar tax and consumption of SSBs in the study group, including identified clusters (%).

Items		Total Sample	Cluster 1 “Unaware of Disease” (N = 41)	Cluster 2 “Moderately Disease-Conscious” (N = 96)	Cluster 3 “Disease-Conscious” (N = 79)	*p*-Value *
Agreement with the statement, “Sugar tax is a good solution in the fight against obesity in Poland”	yes	21.3	22.0	14.6	29.1	0.126
	no	56.9	56.1	58.3	55.7	
	no opinion	21.8	22.0	27.1	15.2	
Familiarity with the sugar tax	0 pts.	13.4	17.1	14.6	10.1	0.840
	1 pt.	33.8	29.3	32.3	38.0	
	2 pts.	37.5	39.0	39.6	34.2	
	3 pts.	15.3	14.6	13.5	17.7	
Frequency of consuming SSBs	less than once a week	50.0	61.0	40.6	55.7	0.041
	once a week or more often	50.0	39.0	59.4	44.3	
**Situations in which SSBs are consumed**						
when meeting friends		63.4	58.5	63.5	65.8	0.734
during events		61.6	56.1	59.4	67.1	0.421
during family celebrations		50.9	43.9	45.8	60.8	0.088
while staying at home		42.6	43.9	45.8	38.0	0.568
while at a restaurant		35.2	31.7	36.5	35.4	0.866
while at university/at work		25.9	22.0	27.1	26.6	0.810
**Reasons for buying SSBs**						
I like their taste		81.5	70.7	82.3	86.1	0.117
this is how I satisfy my thirst		19.5	19.5	30.2	7.6	<0.001
owing to them, I have more energy during the day		12.0	9.8	14.6	10.1	0.588
they reduce my stress/tension		8.3	2.4	8.3	11.4	0.243
they are a reward for my successes		7.9	4.9	8.3	8.9	0.726
they are part of a typical meal for me		5.6	4.9	7.3	3.8	0.591

* Chi^2^ independence test.

**Table 4:** Prediction of sugar-sweetened beverage (SSB) consumption frequency.

Level	SSB Consumption Frequency					
	Less Than Once per Week	At Least Once per Week				
	Ref.	Estimate	Point Estimate (OR)	95% Wald Confidence Limits		*p*-Value
Intercept		−0.9918				0.0226
**Clusters**						
“Unaware of disease”		−0.8283	0.437	0.183	0.941	0.041
“Disease-conscious”		−0.7261	0.484	0.240	0.974	0.041
“Moderately disease-conscious”	Ref.		1			
**Consuming while at a restaurant**						
yes		0.6133	1.847	1.137	3.638	0.046
no	Ref.		1			
**Consuming while at school/work**						
yes		1.1685	3.217	1.505	6.877	0.003
no	Ref.		1			
**Consuming while staying at home**						
yes		1.0568	2.877	1.498	5.526	0.002
no	Ref.		1			
**The reason for consuming is the fact that they like the taste**						
yes		1.5129	4.540	1.656	12.449	0.003
no	Ref.		1			
Familiarity with sugar tax (in points)		−0,5203	0.594	0.416	0.849	0.004

OR—point estimate (β e^β^), 95% confidence intervals.

## Data Availability

The original contributions presented in the study are included in the article, further inquiries can be directed to the corresponding author.
